# An artificial intelligence algorithm is highly accurate for detecting endoscopic features of eosinophilic esophagitis

**DOI:** 10.1038/s41598-022-14605-z

**Published:** 2022-07-01

**Authors:** Christoph Römmele, Robert Mendel, Caroline Barrett, Hans Kiesl, David Rauber, Tobias Rückert, Lisa Kraus, Jakob Heinkele, Christine Dhillon, Bianca Grosser, Friederike Prinz, Julia Wanzl, Carola Fleischmann, Sandra Nagl, Elisabeth Schnoy, Jakob Schlottmann, Evan S. Dellon, Helmut Messmann, Christoph Palm, Alanna Ebigbo

**Affiliations:** 1 (Internal) Medicine III – Gastroenterology, University Hospital of Augsburg, Stenglinstrasse 2, 86156 Augsburg, Germany; 2 Regensburg Medical Image Computing (ReMIC), Ostbayerische Technische Hochschule Regensburg (OTH Regensburg), Regensburg, Germany; 3 Regensburg Center of Health Sciences and Technology, OTH Regensburg, Regensburg, Germany; 4 Center for Esophageal Diseases and Swallowing, Division of Gastroenterology and Hepatology, Department of Medicine, University of North Carolina, Chapel Hill, NC USA; 5 Faculty Computer Science and Mathematics, OTH Regensburg, Regensburg, Germany; 6 General Pathology and Molecular Diagnostics, Medical Faculty, University of Augsburg, Stenglinstrasse 2, 86156 Augsburg, Germany

**Keywords:** Diseases, Gastroenterology, Mathematics and computing

## Abstract

The endoscopic features associated with eosinophilic esophagitis (EoE) may be missed during routine endoscopy. We aimed to develop and evaluate an Artificial Intelligence (AI) algorithm for detecting and quantifying the endoscopic features of EoE in white light images, supplemented by the EoE Endoscopic Reference Score (EREFS). An AI algorithm (AI-EoE) was constructed and trained to differentiate between EoE and normal esophagus using endoscopic white light images extracted from the database of the University Hospital Augsburg. In addition to binary classification, a second algorithm was trained with specific auxiliary branches for each EREFS feature (AI-EoE-EREFS). The AI algorithms were evaluated on an external data set from the University of North Carolina, Chapel Hill (UNC), and compared with the performance of human endoscopists with varying levels of experience. The overall sensitivity, specificity, and accuracy of AI-EoE were 0.93 for all measures, while the AUC was 0.986. With additional auxiliary branches for the EREFS categories, the AI algorithm (AI-EoE-EREFS) performance improved to 0.96, 0.94, 0.95, and 0.992 for sensitivity, specificity, accuracy, and AUC, respectively. AI-EoE and AI-EoE-EREFS performed significantly better than endoscopy beginners and senior fellows on the same set of images. An AI algorithm can be trained to detect and quantify endoscopic features of EoE with excellent performance scores. The addition of the EREFS criteria improved the performance of the AI algorithm, which performed significantly better than endoscopists with a lower or medium experience level.

## Introduction

The incidence of eosinophilic esophagitis (EoE) has risen significantly in the past decade and has become a significant cause of dysphagia and food impaction^1–3^. EoE is diagnosed in the setting of symptoms of esophageal dysfunction and histopathological demonstration of marked esophageal eosinophilia^4,5^. Endoscopic features associated with EoE include edema, rings, exudates, furrows, and strictures^6^. While the presence of these morphological changes is not required for diagnosis, they are supportive and prompt the biopsies necessary for histopathological confirmation^5^. However, the endoscopic features of EoE may be missed, either because physicians are not familiar with them or the morphologic changes are too subtle^7,8^.

The EoE Endoscopic Reference Score (EREFS), based on the endoscopic features described above, has improved the recognition, reporting, and classification of EoE^7,9,10^ but is still not used as a standard tool in many settings^11^. For enhanced detection of EoE, Artificial Intelligence (AI) with deep learning (DL) could be an additional diagnostic option. In general, the application of AI and machine learning (ML) in gastrointestinal (GI) endoscopy has made significant progress in the past few years, especially in the domain of image and pattern recognition^12,13^. Clinical studies have applied AI in benign and malignant disorders with excellent results, including Helicobacter pylori diagnosis, esophageal and gastric cancer, as well as colorectal polyp detection^14–19^. For the diagnosis of EoE, however, there has been only one study to date in which endoscopic images of EoE were assessed using a convolutional neural network (CNN)^20^. EoE was distinguished from the normal esophagus and candidal esophagitis with promising results. In deep learning, CNN architectures use basic convolution modules and complement them with sigmoidal activation functions and pooling operations^21^. In the image-understanding domain, numerous CNN architectures for different tasks have been implemented, allowing for deep networks with 100 layers or more.

With this background, the aims of this study were to develop and then externally validate a deep learning-based AI model to detect EoE and quantify EREFS and assess the ability to recognize endoscopic images of EoE and report EREFS of human endoscopists as compared to the AI model.

## Methods

This was a 3-phase study in which an AI model was trained to detect EoE on endoscopic white light images. In the first phase, the AI model was trained and validated with an internal data set (InD). In the second phase, the performance of the AI model was tested on an external data set (ExD) from a separate hospital; in this phase, the benefit of using the EREFS scores in the AI model was studied. In the third phase, the performance of the AI model was compared with human endoscopists with different levels of experience.

### Data and image acquisition

The pathology reports archived in the laboratory information system (Nexus, Frankfurt a.M, Germany) of the Institute of Pathology and Molecular Diagnostics of the University Hospital of Augsburg, Germany, were screened for the german terms “Ösophagus” and “eosinophile Ösophagitis”. The corresponding endoscopic reports and white light images of patients identified within a 10-year period between 06/2010 and 05/2020 were extracted from the endoscopy database (Viewpoint 5, GE Healthcare Systems (Germany)) of the University Hospital of Augsburg, Germany, by two board-certified gastroenterologists. Endoscopic images were selected for AI training according to the following criteria:

(1) Inclusion criteria:

- Images from patients with active EoE (≥ 15 eosinophils/HPF) who were diagnosed as per consensus guidelines^5^
- Images from patients with an endoscopically normal-appearing esophagus who also had normal esophageal biopsies

(2) Exclusion criteria:

- Images with other visible pathologies, such as reflux esophagitis, candida esophagitis, mass, or other findings
- Images with visible stricture formation and stenosis
- Poor quality images with blurring, inadequate focus, excessive bubbles, blood or mucus covering the mucosa

All images of the InD were taken with an Olympus gastroscope (GIF-HQ190, GIF-HQ-180; Olympus Medical Systems, Tokyo, Japan) at the University Hospital Augsburg, Germany.

### EREFS

The images were assessed for the EREFS by two board-certified gastroenterologists. EREFS were reported using the standard scoring system, including edema 0–1 point, rings 0–3 points, exudates 0–2 points, and furrows 0–2 points^7,10^. Images with obvious strictures were excluded (total score range, 0–8) because it was assumed that the additional benefit of AI support in patients with stricture formation or stenosis is limited, and the actual challenge lies in the identification of EoE patients with more subtle endoscopic features, who are probably in an earlier phase of the disease.

In addition to the main binary classification branch (EoE vs. normal), a specific auxiliary branch for each of the EREFS categories was included in the training phase of the AI system. In other words, two AI models were trained, one with (AI-EoE-EREFS) and a second without the auxiliary EREFS categories (AI-EoE).

### AI-model construction and training

The training of both AI models was based on a CNN with a ResNet architecture^22^. The models were pretrained on a non-medical dataset (ImageNet^23^) to learn basic abstract visual features. The final classification layer of the neural network was then adjusted to enable a binary classification—the detection and classification of EoE. The threshold probability was set to 0.5. Before training, InD images were cropped to exclude black borders and resized for consistency across the dataset, after which data augmentation, including scaling and shifting of images, was applied. The intention of data augmentation was to enable the algorithm to be more robust to slight variations in the input images. During training, the model’s parameters were optimized to minimize the cross-entropy loss with label smoothing, achieve a global binary prediction, and accurately classify the particular EREFS features. The models were trained for 6000 iterations with a batch size of 48 and a sampling strategy such that both classes are equally represented in each batch. The initial learning and weight-decay for the Stochastic Gradient Descent algorithm were set to 0.01 and 5e−4. Over the course of training, the learning rate was decayed with a cosine annealing schedule. All models were implemented in the PyTorch Deep-Learning framework.

### Internal validation

To internally validate the models, we performed five repeated runs of five-fold cross-validation. In five-fold cross-validation, the dataset is split into five disjoint subsets. Four of the five folds are used as training data for the algorithm. The remaining one is the held-out validation set. The procedure is repeated such that each fold was in the role of the validation set once. We did not perform hyperparameter optimization or early stopping techniques on the validation set but trained our algorithms for a fixed number of iterations. The cross-validation scheme is repeated five times with randomized subset compositions and seeds for the random number generators from 0 to 4.

### Test set with external data

**Figure 1:**
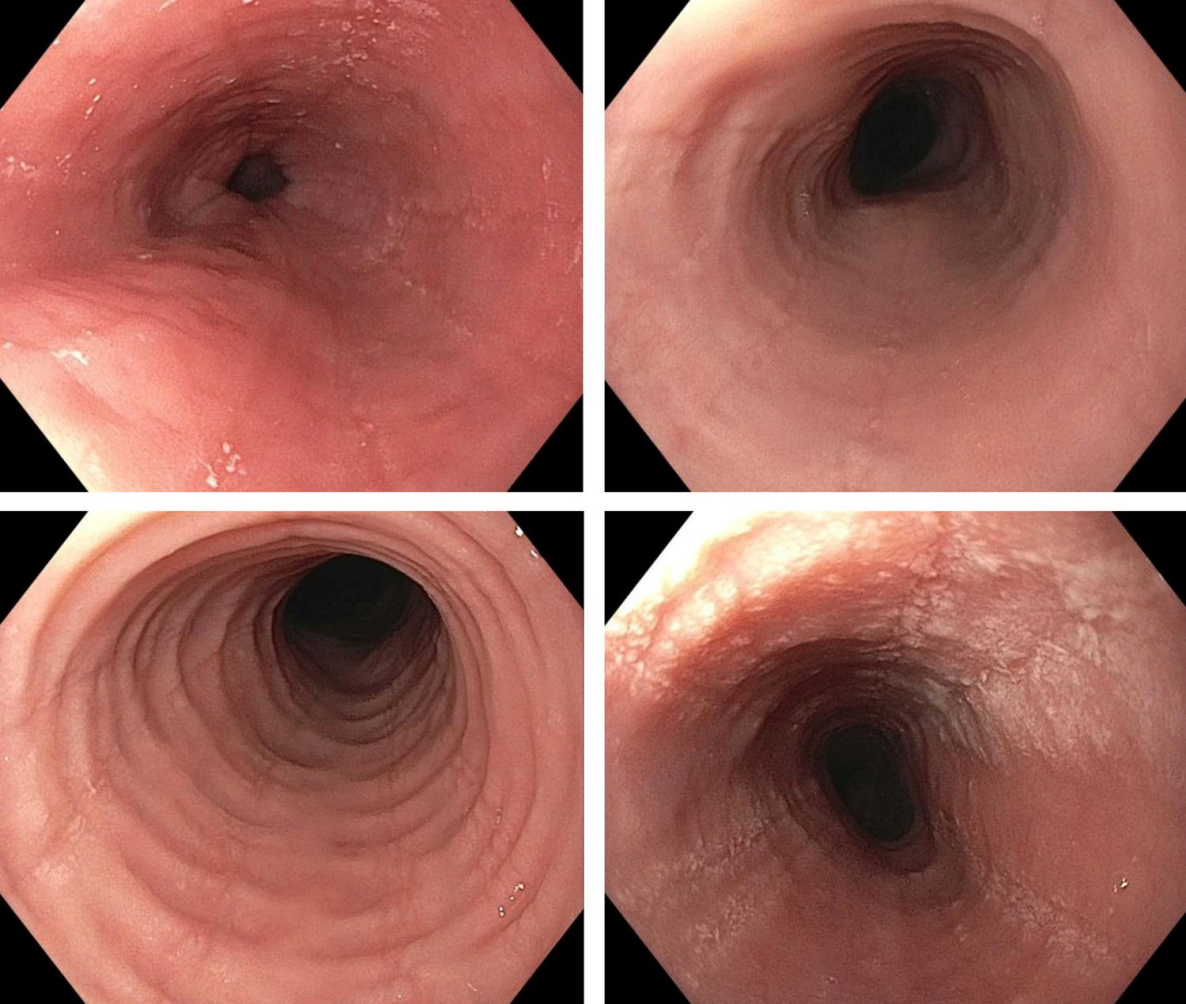
Endoscopic white light images of eosinophilic esophagitis showing furrows, exudates, edema, and rings.

**Figure 2:**
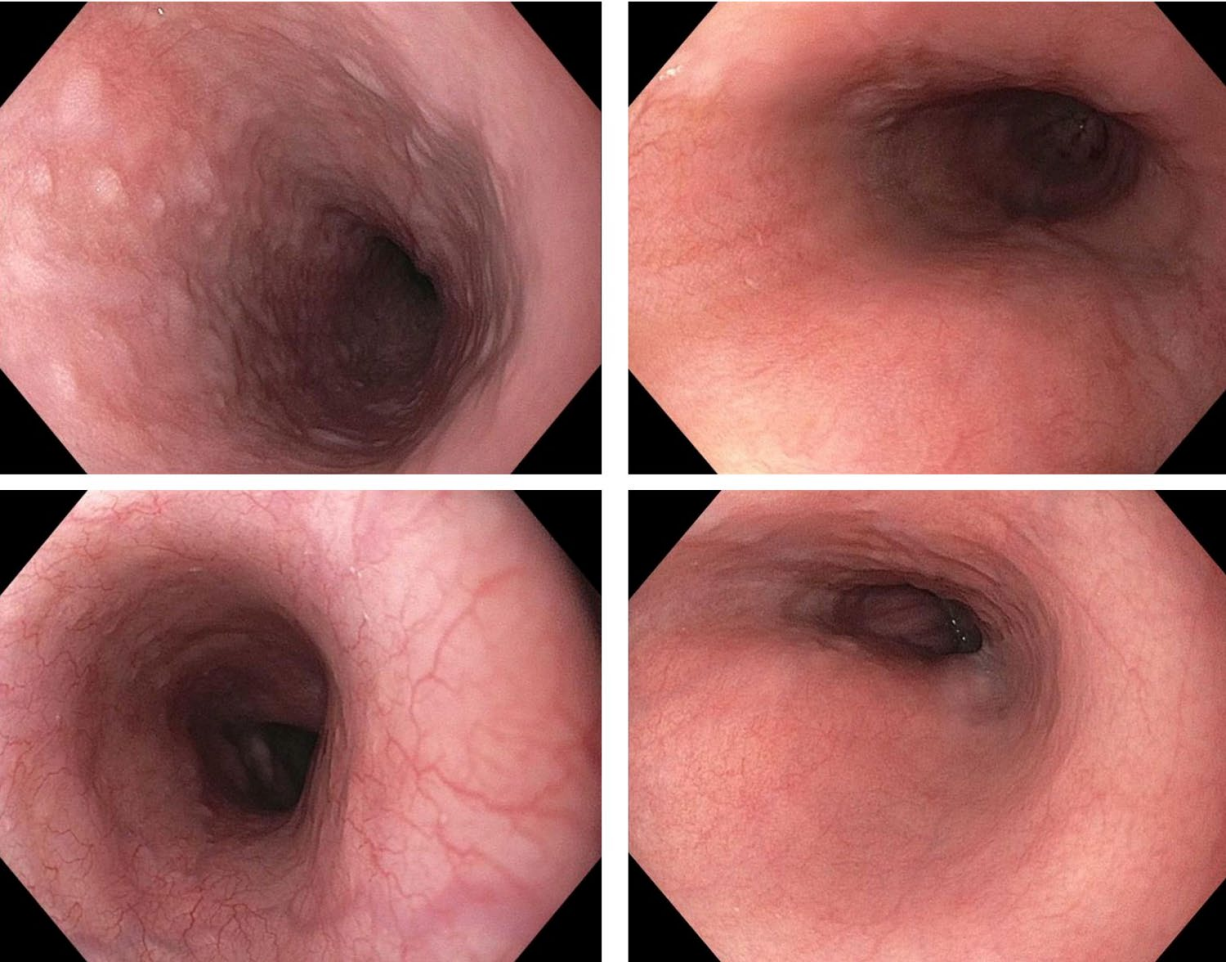
Endoscopic white light images of a normal esophagus.

After constructing the AI models, we evaluated their performance on an independent and externally acquired test set (ExD). ExD comprised a total of 200 WL images, including 100 WL images from EoE patients with active disease (≥ 15 eos/hpf) diagnosed per consensus guidelines and 100 WL images of normal esophagus in patients without any visible, histologic, or known esophageal pathology. The test set was provided by the University of North Carolina, Chapel Hill (UNC), with patients who underwent endoscopy between August, 2020, and January, 2021. Both AI algorithms had never seen the ExD images before the evaluation. The evaluation and analyses for these images were performed in a blinded fashion, with the code of EoE vs. normal only revealed after the results of AI-EoE and AI-EoE-EREFS had been finalized and transmitted to the UNC. Exemplary images are shown in Figs. 1 and 2. For the external evaluation, an ensemble of the five individual models from the first cross-validation run was employed.

### Image evaluation by endoscopists

To better understand the performance of AI-EoE and the impact of EREFS on the diagnostic accuracy, ExD images were evaluated by six endoscopists who were rated according to their level of experience, including:

1. Endoscopy beginners (n = 2)
2. Senior fellows (n = 2)
3. Consultant endoscopists (n = 2)

Endoscopists were asked to assess the images for the presence of EoE according to the following process:

### Group 1

Assessment of all 200 ExD images (1–200) according to the clinical impression of the endoscopist after looking at the images without explicit use of EREFS.

### Group 2

Assessment of the first 100 ExD images (1–100) according to the clinical impression of the endoscopist. After this, the endoscopists were asked to review the initial description of the EREFS criteria by Hirano et al.^10^; they were also shown 30 representative endoscopic white light images of EoE with the corresponding EREFS scores. Following this training phase, an additional assessment of the second 100 images (101–200) using the EREF score was performed. The assessment of the first 100 images was done for adjusting the individual performance of the endoscopists. The assessment of the second 100 images was done to quantify the enhancement of diagnosis having the EREFS explicitly in mind. Each group contained one endoscopist from each experience level.

### Statistical analysis and outcome measures

The sensitivity, specificity, accuracy, the area under the ROC curve (AUC), and the harmonic mean (F1) between sensitivity and precision on the ExD images were used to measure the performance of the models, AI-EoE and AI-EoE-EREFS, trained without and with the additional EREFS branches, respectively. These statistics are calculated from the true positives (TP), false positives (FP), true negatives (TN), and false negatives (FN) produced by the algorithm.

$$ {\text{Harmonic}}\;{\text{mean}}\;\left( {{\text{F1}}} \right) = {\text{2TP}}/\left( {{\text{2TP}} + {\text{FP}} + {\text{FN}}} \right) $$

$$ {\text{Sensitivity}} = {\text{TP}}/\left( {{\text{TP }} + {\text{ FN}}} \right) $$

$$ {\text{Specificity}} = {\text{TN}}/\left( {{\text{TN}} + {\text{FP}}} \right) $$

$$ {\text{Accuracy}} = \left( {{\text{TP}} + {\text{TN}}} \right)/\left( {{\text{TP}} + {\text{TN}} + {\text{FP}} + {\text{FN}}} \right). $$

Statistical significance between groups was determined with the McNemar test.

By testing multiple models, we investigated whether the inclusion of the EREFS criteria led to an improvement in the performance of AI-EoE.

The performance of the human endoscopists on the same data set (ExD) was also evaluated using the same parameters described above.

### Ethics

Ethics approval was granted by the Institutional Review Board of the University Hospital Augsburg (BKF Nr. CCE03022021_0002, date: 04/07/2020), as well as by the Institutional Review Board of UNC (number 20-3655; date of initial approval: Jan 28, 2021). All methods used in this study were carried out in accordance with the declaration of Helsinki and in accordance with relevant guidelines and regulations. All images used in this study were obtained from endoscopic procedures for which patients had provided their informed consent. For patients under 16 years, parents or legally authorized representatives provided informed consent.

## Results

A total of 401 images of EoE from 61 patients and 871 images of a normal esophagus from 393 patients were used to internally train and validate the AI models. The baseline characteristics of patients are shown in Table 1. The distribution of the EREFS on the InD images with EoE was 0–3 (n = 303), 4–6 (n = 98), and 7–8 (n = 0) with a mean EREFS of 3.1 and standard deviation of 0.89.

**Table 1:** Baseline characteristics of patients whose images were included in the study.

	EoE n = 61	Control n = 393	P
Age (mean ± standard deviation)	35.1 ± 19	31.9 ± 25	0.039
Sex (Male/Female)	39/22 (64%/36%)	180/213 (46%/54%)	0.006
**Symptoms**			
Dysphagia	37 (61%)	65 (17%)	0.000

### Performance of AI-EoE and AI-EoE-EREFS on the internal data

In the internal validation, the mean scores and standard deviations achieved with the AI-EoE model for sensitivity, specificity, accuracy and harmonic mean (F1) were: 0.857 (0.016), 0.959 (0.007), 0.927 (0.003), 0.881 (0.005).

The results for the AI-EoE-EREFS algorithm for the respective metrics were: 0.866 (0.006), 0.957 (0.007), 0.928 (0.005), 0.884 (0.005).

**Figure 3:**
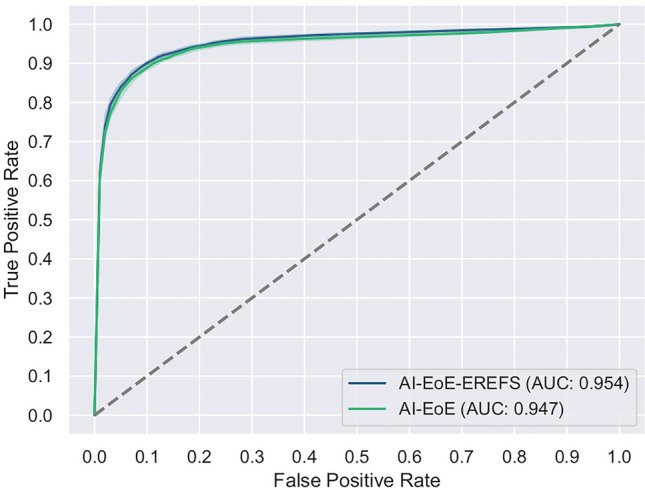
ROC curves and AUC values of AI-EoE and AI-EREFS on the internal data set (InD).

The AUC values for AI-EoE and AI-EoE-EREFS were 0.947 and 0.954, respectively (Fig. 3).

There was no significant difference between AI-EoE and AI-EoE-EREFS in the internal validation using cross-validation.

### External validation: performance of AI-EoE on the external data set (ExD)

The overall sensitivity, specificity, accuracy, and F1 of AI-EoE were 0.93 for all measures (Table 2). The AUC for AI-EoE was 0.986.

**Table 2:** Performance of human endoscopists and AI- models in diagnosing eosinophilic esophagitis on endoscopic white light images.

	Group 1			Group 2			Overall
	1–100	101–200	all data	1–100	101–200 (after EREFS training)	All data	All data and both groups
**Beginner**							
Sens	0.46	0.66	0.56	0.40	0.58	0.49	0.53
Spec	1.00	0.94	0.97	0.46	0.96	0.71	0.84
Accuracy	0.73	0.80	0.77	0.43	0.77	0.66	0.68
F1	0.63	0.77	0.70	0.41	0.72	0.55	0.63
**Fellow**							
Sens	0.90	0.84	0.87	0.60	0.66	0.63	0.75
Spec	0.96	0.98	0.97	0.92	0.98	0.95	0.96
Accuracy	0.93	0.91	0.92	0.76	0.82	0.79	0.86
F1	0.93	0.90	0.92	0.71	0.79	0.75	0.83
**Consultant**							
Sens	0.94	0.98	0.96	0.88	0.98	0.93	0.95
Spec	1.00	0.98	0.99	0.68	0.52	0.60	0.80
Accuracy	0.97	0.98	0.97	0.78	0.75	0.77	0.87
F1	0.97	0.98	0.97	0.80	0.80	0.80	0.89

	AI-EoE			AI-EoE-EREFS			
	1–100	101–200	All data	1–100	101–200	All data	
Sens	0.96	0.90	0.93	0.98	0.94	0.96	
Spec	0.94	0.92	0.93	0.94	0.94	0.94	
Accuracy	0.95	0.91	0.93	0.96	0.94	0.95	
F1	0.95	0.91	0.93	0.96	0.94	0.95	

Group 1 endoscopists relied on their clinical experience, while Group 2 was educated on the EREFS criteria for the second batch of images. AI-EoE was trained with binary classification, while AI-EoE-EREFS was trained additionally using auxiliary branches generated from the EREFS scores.

### External validation: performance of AI-EoE-EREFS on the external data set (ExD) (Table 2)

The sensitivity, specificity, accuracy, and F1 of AI-EoE-EREFS were 0.96, 0.94, 0.95, and 0.95, respectively (Table 2). The AUC for AI-EoE-EREFS was 0.992.

While the AI-EoE-EREFS was numerically superior to AI-EoE, the performance did not differ significantly.

### Performance of endoscopists on ExD data set (Table 2)

Six endoscopists (three per group) assessed the ExD images as follows:

#### Group 1 (ExD images 1–200 based solely on image review)

*The endoscopy* beginner, senior fellow and consultant endoscopist had an overall sensitivity, specificity, accuracy, and F1 of 0.56, 0.97, 0.77 and 0.70; 0.87, 0.97, 0.92 and 0.92, as well as 0.96, 0.99, 0.97 and 0.97, respectively. Accuracy and F1 differed by + 10% and + 22%, respectively, for the beginner, − 2% and − 3%, respectively, for the senior fellow and + 1% and + 1%, respectively, for the consultant endoscopist regarding the first 100 images and the second 100 images.

#### Group 2 (ExD images 1–100 based solely on image review; ExD images 101–200 based on EREFS)

On the first 100 images, the endoscopy beginner, senior fellow and consultant endoscopist had a sensitivity, specificity, accuracy, and F1 of 0.40, 0.46, 0.43 and 0.41; 0.60, 0.92, 0.76 and 0.71, as well as 0.88, 0.68, 0.78 and 0.80, respectively. After education and information on the EREFS scores, the endoscopy beginner, senior fellow and consultant endoscopist improved their performance with a sensitivity, specificity, accuracy, and F1 of 0.58, 0.96, 0.77, and 0.72; 0.66, 0.98, 0.82, and 0.79, as well as 0.98, 0.52, 0.75, and 0.80, respectively. Therefore, the accuracy and F1 differed by + 79% and + 76%, respectively, for the beginner, + 8% and + 11%, respectively, for the senior fellow and − 4% and 0%, respectively, for the consultant endoscopist regarding the first 100 images and the second 100 images.

### Comparison of endoscopists with the AI models

**Figure 4:**
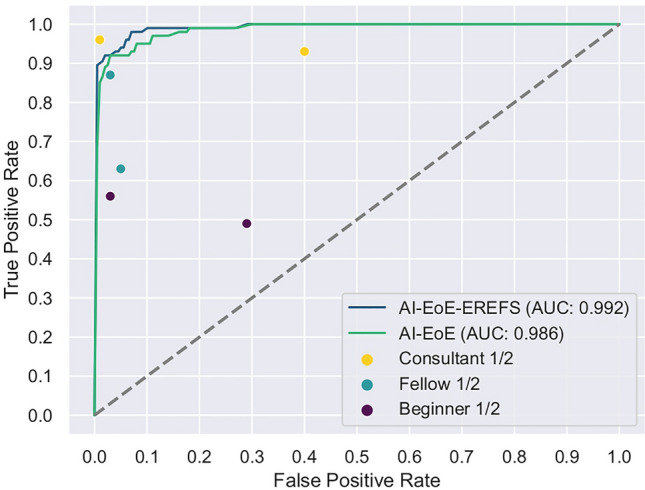
ROC curves and AUC values of AI-EoE and AI-EoE-EREFS on the external data set (ExD) compared with human endoscopists who had varying experience levels.

The overall performance of the AI models with and without auxiliary EREFS was better than the performance of the beginners, senior fellows, and consultant endoscopists. Using the McNemar test, we found a statistically significant difference in the accuracy and sensitivity between AI and beginners. The AI-EoE-EREFS algorithm also shows statistically significant improvements in sensitivity over the senior fellow group. The specificity did not improve significantly for both models. In the comparison between AI and consultant endoscopists, no significant difference could be found. The ROC curve comparing human endoscopists with AI is shown in Fig. 4; the AUC of AI-EoE and AI-EoE-EREFS was 0.9862 and 0.9924, respectively.

## Discussion

EoE is becoming increasingly important due to its rising incidence, but endoscopists may still have difficulty detecting and diagnosing EoE during routine EGD, leading to diagnostic delay^1,2,24,25^. The suspicion of EoE during routine EGD and based on the endoscopic image alone is challenging^7,8^, and the presence of EoE must be suspected either clinically or macroscopically to prompt taking esophageal biopsies. A study from Denmark illustrates that even in dysphagia patients, adequate biopsy sampling is often not performed^26^. With the advancement of AI and ML, assistance can be offered to endoscopists in various detection and characterization of pathologies. A first paper on the use of AI in the detection of EOE demonstrated excellent sensitivity and specificity on an internally validated image dataset^20^. Guimarães et al. were able to show an overall accuracy of 0.91 in their study. Interestingly, the CNN model was also able to distinguish esophageal candidiasis, which has white plaques representing an important differential diagnosis of EoE during EGD. This shows the enormous potential of AI and especially deep learning.

Our study sought to demonstrate the robustness of a trained CNN model by additionally evaluating and validating its performance on externally acquired data. Testing on external data is essential because AI models should work for and generalize towards new data to avoid overfitting bias^27^. On external endoscopic WL images, our AI model, which was trained not only with a binary classification but also with the auxiliary EREFS data, produced an overall sensitivity, specificity, and accuracy of 0.96, 0.94, and 0.95 in the detection of EoE. It must be stated that the results of the external validation were even better than the internal cross-validation because the internal validation was done without additional hyperparameter tuning.

**Figure 5:**
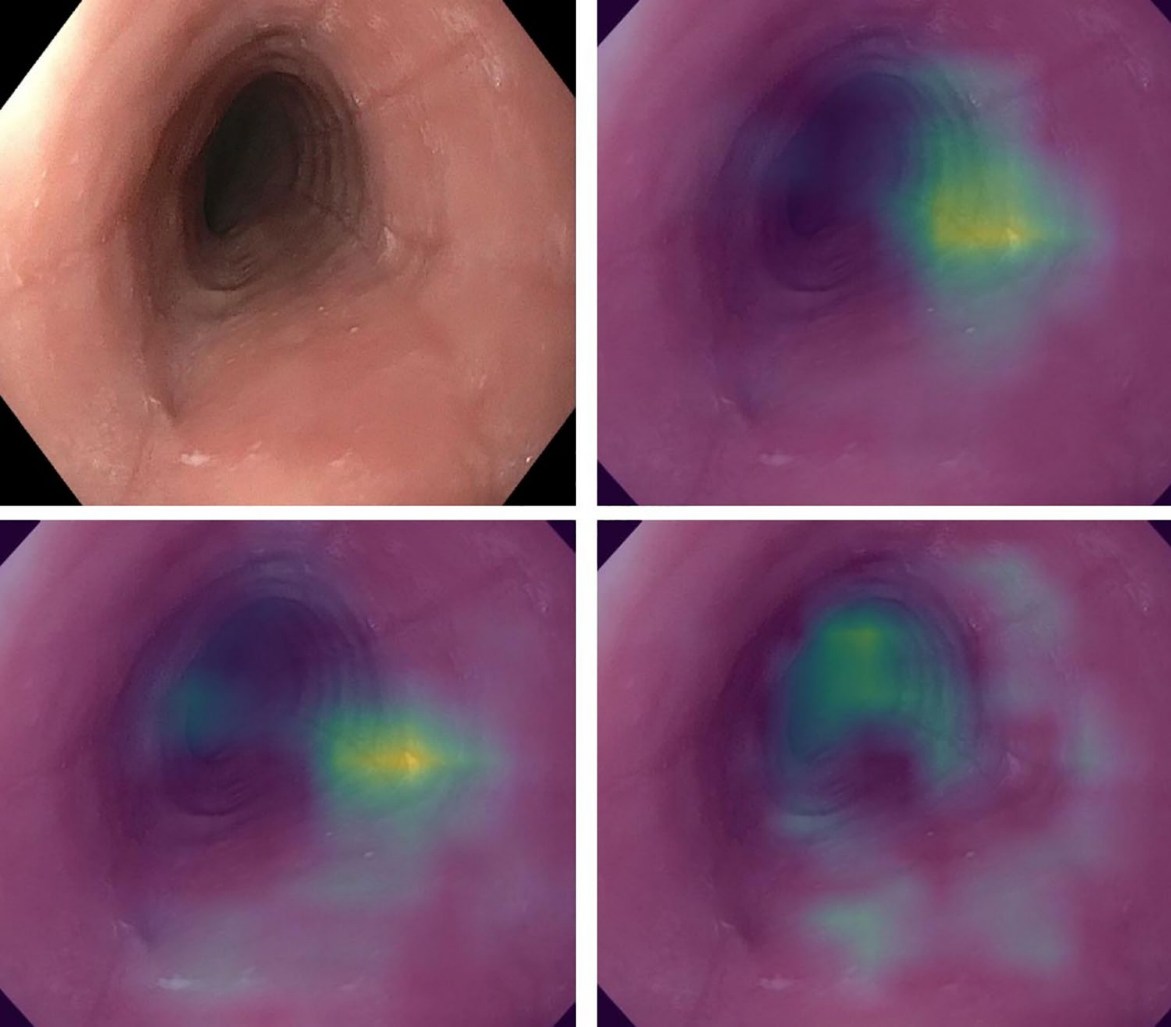
Features detected on input images by AI-EoE-EREFS are highlighted using Gradient-based visualization (Grad-CAM)^28^: the top left image shows the original endoscopic image with furrows, exudates, and rings; in the top right image, furrows are highlighted, while in the bottom left and bottom right images, exudates, and rings are highlighted, respectively.

Interestingly, the addition of the auxiliary EREFS categories (AI-EoE-EREFS) improved the performance of the AI model. Grad-Cam visualizations (Gradient-weighted Class Activation Mapping) point to the inclusion of EREFS, forcing the model to detect features in the input that more cleanly align with the human understanding of the image (Fig. 5)^28^. The visualizations in combination with the improved metrics hint that augmenting the network with EREFS is beneficial, although we could not prove this result with statistical significance in this study.

The EREFS system was developed and validated to improve and standardize the reporting of endoscopic features of EoE, but also to aid with diagnosis and monitoring, both in practice and in trials^9,10,29–33^. For endoscopists, being aware of the EREFS criteria may lower the threshold of biopsy sampling in the esophagus, which could subsequently lead to higher sensitivity for EoE during routine EGD. An essential aspect of this may also be the experience of the physician performing EGD. We divided the participating endoscopists into two groups to investigate whether the EREFS categories also affected their performance. Group 1 assessed all ExD images based on their clinical impression and experience. Group 2 was not reminded of the EREFS criteria and was also asked to assess the first 100 images based on their clinical impression. After this, physicians in group 2 were educated on the EREFS criteria and then asked to evaluate the second batch of ExD images. Similar to the AI model, the EREFS criteria improved the beginner endoscopists' performance as well as the senior fellows’ performance in this process. In our experiment, the performance improved for the beginners using EREFS, adjusted to the baseline performance of the individual for accuracy and F1, by 69 and 54 percentage points. Even senior fellows profited, given the improvement in accuracy and F1 by 10 and 14 percentage points, respectively. These results suggest that the training of endoscopists with the EREFS scores, especially beginners, can improve their diagnostic accuracy for eosinophilic esophagitis considerably. Eluri et al. showed that the proportion of patients with a normal esophagus decreased significantly after the introduction of the first EoE guidelines, suggesting improved recognition of endoscopic findings with the EREFS criteria^7^. The overall performance of the endoscopy beginners and senior fellows was well below the more experienced consultant endoscopists. Comparing the overall results for all data of the group of beginners, senior fellows, and consultant endoscopists with the AI results, AI performs best, irrespective of whether the EREFS criteria were used or not. However, AI models may be particularly suitable for the support of endoscopists with lesser experience. In addition, the training and education of beginners may improve with the help of an AI model.

As with most studies showing the construction and validation of endoscopic AI models, the major limitation of this study is the amount and diversity of data available for training and validation. In addition, the number of endoscopists who underwent evaluation of the dataset was small, thereby limiting the validity of the comparison to the human endoscopists. Also, the fact that data was collected retrospectively may have influenced the quality of the proposed algorithm. Nevertheless, the excellent results on the test data set may show that the generalizability of the AI model was sufficient, at least on the images provided by the external partners. Another limitation is that only a two-class differentiation, EoE vs. normal esophagus, was done. This may not depict the real-life situation sufficiently, where other pathologies, such as reflux esophagitis, candida esophagitis, lymphocytic esophagitis, lichen planus, and others, must be considered. Finally, even though our first step was to use static endoscopic images, video images, or even real-time assessment during EGD will be necessary to assess for this AI tool to be used in clinical situations. In real-life, blurry images, bubble formation, and even blood and mucus may influence the quality of vision, making it difficult to detect and diagnose pathologies such as EoE.

In conclusion, our study showed that an AI model can be developed to identify EoE using endoscopic still images and validated from an external data set with excellent performance. Additional training with the EREFS scores improves the diagnostic ability of human endoscopists as well as the AI model. Future directions will incorporate video so that this tool can ultimately be used at the point of care.

## Data Availability

The datasets used and/or analysed during the current study are available from the corresponding author on reasonable request.

## Abbreviations

Acc: Accuracy
AI: Artificial intelligence
CNN: Convolutional neural network
DL: Deep learning
EoE: Eosinophilic esophagitis
EREFS: EoE Endoscopic Reference Score
ExD: External data
InD: Internal data
SE: Sensitivity
SP: Specificity
WL: White light
